# Intelligence

**Published:** 1827-09

**Authors:** 


### INTELLIGENCE.

### MONTHLY REPORT OF PREVALENT DISEASES.

During the early part of the time embraced in the present Report, the heat of the weather was considerable, and was attended by a corresponding increase in the number of cases of biliary derangement. Although these generally assumed the form of diarrhœa, in some instances they passed into cholera; but decided cases of this nature have been much less frequent than during the corresponding portion of last year. The vicissitudes of the atmospheric temperature have been considerable, in consequence of which acute inflammatory attacks have been more frequently met with than is customary at this season, and the chronic pulmonary diseases, which often intermit during the summer, have not undergone their usual degree of mitigation.

*New Cupping Glass.*—There is at present in the shop of Mr. Millikin, surgical instrument maker in the Strand, a novel kind of cupping glass, contrived by Dr. Barry. It is intended to remedy the difficulty lately experienced at Guy's Hospital, in a case of hydrophobia, where, from the situation of the wounds, on and between the fingers, the ordinary cupping glass could not be applied.

The novelty consists in the glass being large enough to receive the whole hand, and having a bottle of Indian rubber attached to the rim, and sufficiently opened at its bottom to admit (with some difficulty) the hand, and afterwards to embrace closely the wrist and part of the forearm. When the common exhausting syringe is applied to this glass, the effect upon the contained hand is very powerful. Our correspondent has no hesitation in expressing a favourable opinion of this simple improvement, as it affords the advantages both of the ligature and cupping glass, so much insisted upon by M. Magendie and others.

*Stimulating Applications to the Ear.*—Sir, Last December, a gentleman, whose case I had declared was nearly or quite incurable, called on me from one of our oldest and most respectable surgeons, requesting to see me. On visiting him, that gentleman proposed that I should use the ointment of nitrate of mercury, which he said was different from that commonly made, by being supersaturated with nitric acid, and was still further improved from being kept several years; and, as it was impossible to get any possessing this latter quality elsewhere, he gave me a piece of some he had by him. It was applied according to his wish, after I had stated to my patient, that I would neither claim merit nor take disgrace from the experiment, and after a short trial, during which the meatus became excoriated (for it was applied to one side only), the patient had the ointment from me, and applied it profusely. At the end of four days, he became one morning (according to his own description) as if under the influence of night-mare, and in attempting to speak, though his mind willed certain words, yet the motary powers of the tongue refused their office, and he uttered incoherent nonsense; in a few minutes he ceased to have the power to speak, yet had his senses perfectly in other respects. Cupping, bleeding, and active antiphlogistic treatment restored him; but he has since experienced at times the most distressing noises, or false perceptions, which appear to be external,—such as hearing an organ under his window, hounds in cry, horses galloping, and more constantly he fancies he hears the crowing of a cock, who, if beginning his note when the patient loses all recollection in sleep, seems to continue and finish it when he awakes. By inducing and keeping up a regularity of the bowels, and by the use of tonic medicine, these sensations are less distressing, yet still sufficiently so to caution practitioners against the use of this ointment, which is much too commonly prescribed.

I am, Sir, your obedient servant,

W. WRIGHT.

15, *Princes-street*, *Hanover-square*; *July* 1827.

---

*College of Physicians versus* Dr. HARRISON.

SIR,—In a late Number of your Journal, you published the correspondence between Dr. HARRISON and Dr. CHAMBERS, and appended to it some observations of your own, calculated to urge the College to try the question at law with Dr. Harrison, on the double plea—first, that, without such trial, and enforcement of power, existing Licentiates were not duly protected in privileges for which they had paid down their money; and secondly, that physicians, intending hereafter to practise in London, would, without such an exercise of authority, be emboldened to set the College at defiance altogether. Whether urged by such representations, or by their own sense of duty, I know not, but it appears that the tocsin of war has been sounded. Dr. Harrison is currently reported to have received notice of action. Sir J. Scarlett and Mr. Brougham have got their retaining fees for the College, and solicitors on both sides are giving dreadful note of preparation. Under these circumstances, painful to contemplate, permit a bystander to offer a little disinterested advice to all parties,—to the President and Fellows,—to Dr. Harrison, who braves their authority,—and to yourself.

The view, Sir, which you have taken of this question appears to me to be singularly unfortunate. With an apparent, and (I may safely add) most sincere desire to be impartial, your remarks were in reality calculated to foment discord, and to close the avenue to professional harmony. You have overstrained one argument, and shut your eyes to others, and in this way have led to some very erroneous impressions, which it is the aim of this letter to refute. My object, I again repeat, is peace and harmony in the profession. Bickerings among professional men are always viewed with great disfavour by the public; and the provocation must be strong indeed, and the benefit to be gained clear and substantial, before the public mind will acknowledge the justice of checking a man in the exercise of his calling.

That Dr. Harrison's letter to Dr. Chambers was most intemperate, ill-judged, and unwarranted, most persons, I presume, will at once admit. That Dr. Chambers was perfectly justified in refusing to meet Dr. Harrison cannot be disputed for a moment. His answer was such as became him, and Dr. Harrison's observations in reply are pettish, and in very bad taste. All this I admit; but then comes the question, is this hasty and unwise conduct to be made the groundwork of a serious proceeding in law? Is it reckoned prudent or wise in a general to give battle, because some Goliath comes out in front of the army, and acts the bravado? Let us meet this question fairly. If the College of Physicians perceive an increasing disinclination on the part of physicians (not being English graduates) to come before them,—if the number of physicians practising in London without licence from them be increasing,—if evil be thereby springing up to the king's lieges, then is there indeed a *nodus vindice dignus.* It then behoves the College to bestir themselves, to show that they have at heart the interest of the community, and are deserving of the privileges they enjoy. But, if they find this a solitary (or nearly solitary) instance of defiance,—if the number of Licentiates be fully equal to those of former years,—if the conduct of Dr. Harrison be generally disapproved of,—if there still remain ample reasons for believing that the interest, no less than the good sense, of physicians will bring them before the President and Censors in time to come as in times past,—then do I say, Sir, that the College will better show their prudence in abstaining from, than in courting the publicity of, a trial at law.

Now, Sir, I maintain that such is the exact position of the College at this moment. Their authority is acknowledged by all the respectable physicians of London. The public attaches a high degree of importance to the title of "Member" or "Licentiate" of the College; and this alone would be a sufficient motive with very many. But, far more than all, the qualification of a Licentiate is indispensable to all aspirants to the public hospitals and dispensaries of London. This it would still be, though the College were to lose their action to-morrow; and I feel the most perfect confidence that, even in such an event, (which, however, I do not anticipate,) the same number of candidates for the College licence would still annually appear before them. The College ought to know, and I hope they do know, that their truest and noblest source of power is—not the charter of Henry the VIIIth, but the charter of public opinion. They enjoy the good opinion of the public for many reasons, but mainly for this, that they have run an even and quiet course,—that their influence has been gained by time and in silence, and not by sudden displays of energy,—that they have not obtruded their privileges upon the public eye, nor used the power that has descended to them from very distant times with any thing like harshness or petulance. My earnest advice to them is—not to swerve from the path which has hitherto proved so fortunate for them, and so useful to the public,—not to allow themselves to be hurried away by the bold bearing of one angry man to an act that savours more of bravery than of caution.

Let us imagine for a moment that my advice is taken, that Dr. Harrison's sins (supposing them to be such) are overlooked, in what respect is the College worse off than they were? He has been practising for at least ten years in London. If zeal for the good of the public induces them to prosecute, the public will feel and say that they ought to have done so long ago. They will "take nothing," as the lawyers say, by that motion. If a desire to do justice to existing Licentiates actuate them, I, as one, beg to say that I require no such stretch of kindness. I am perfectly satisfied with what I have got for my money, partly in increased respectability, partly in increased notoriety, but chiefly in the entrance it has afforded me to two charitable institutions.

But, Sir, supposing that my advice is not taken, that Dr. Harrison's challenge is accepted, the action entered for trial in Westminster Hall, and the lawyers in their wigs are arranged "in terrible show;" it will, I presume, be conceded to me that the verdict will either be for plaintiff or defendant. If Dr. Harrison gains his action, either from a flaw in the indictment (a very conceivable case) or on the merits, the consequences are serious. I will not stop to investigate them, because I expect that the College will carry their point, and compel Dr. Harrison to pay them 5*l.* a month. What will be the feeling of the public in such an event on this subject? They will probably reason in the following manner:—The College have carried their point: "the law allows it, and the court awards it." But is not the College of Surgeons in as high esteem as the College of Physicians, and do they enjoy such a privilege? Is it not a sufficient penalty to be debarred from the privilege of meeting your professional brethren in open day,—to be denied access to the principal hospitals and dispensaries of the metropolis,—to be found wanting in that list of "learned, grave, and profound practisers in the faculty of physic," which is annually put forth for the guidance of the lieges in their choice of a doctor? Is not all this enough, without the penalty of 5*l.* a month?

Besides, though this be law, where is the equity of the power thus vested in the College? Beyond the seventh mile-stone from the standard in Cornhill, Dr. Harrison, and Drs. A. B. and C., are legally entitled to practise physic every hour of their lives; and though Dr. Chambers very properly refused to meet Dr. Harrison in consultation either at Brompton or Fulham, he could hardly refuse to meet him at Wandsworth, and certainly not at Richmond. The public would not see much reason in this. It has been attempted to defend this privilege on the ground that the influx of severe cases into London requires that additional safeguards should here be thrown about the lives of individuals. This argument, however, we may safely set aside as sheer nonsense. It is perfectly clear that the diploma of the university of Edinburgh, if sufficient to authorise a man in the practice of physic at Liverpool, (which no one questions,) is, in equity, quite sufficient for London. But the College have a charter, confirmed by Act of Parliament, and that charter gives an additional security to those whom good fortune has fixed either in or within seven miles of London. But the public will not overlook this important fact, that a physician practising in London *is not practising without licence*, for his diploma gives him authority "medicinam colendi docendi et faciendi, hic, et *ubique terrarum*." His licence is a general one, and general rules must bend to particular circumstances. The public would see this, but they might happen to think at the same time that, if a charter was now for the first time to be granted to the College of Physicians, it would probably resemble that recently given to the College of Surgeons. The honour of being enrolled in it, and the discredit of exclusion, would doubtless be considered sufficient inducements to swell the ranks both of Fellows and Licentiates, without any mention of fine; and so it would, I am well convinced. A few independents would now and then start up, but the great majority of the practitioners of physic in and about London would be enrolled, under some denomination or another, in such a College.

Here I conclude. My argument may be summed up in a few words, though I think none of your readers can well have mistaken it. I acknowledge the power of the College. I believe that, if they persevere, they will gain their action; but, in so doing, I aver—first, that they will incur some risk, and perhaps a little odium, without adequate *public* grounds;—secondly, that the real and best source of their power is the good opinion of the public, which may be shaken, but cannot be increased by a public trial;—thirdly, that there is not the smallest fear of Dr. Harrison's example becoming contagious;—fourthly and lastly, that, if the College are really anxious to try the question of their right to summon before them, and to fine for contumacy, they had better take some other opportunity, rather than be *driven* hastily into the measure by the idle vaunting of an individual, who, in a moment of irritation, throws down the gauntlet of defiance. The charter of the College, though old-fashioned, has hitherto worked well. Let the College think seriously ere they bend the old bow to its full stretch of power. By precipitate opposition, they may unwarily be loosening some of the less apparent but most honourable foundations of their power, and, while they triumph over Dr. Harrison, excite among those who are always but too forward on such an occasion, the popular cries of Persecution and Monopoly.

I am, Sir, your obedient humble servant,

A LICENTIATE OF THE LONDON COLLEGE.

*August 2, 1827.*

*** It was our intention to have made some observations on the preceding letter, but, as we are pressed for room, we must postpone them.

---

*University of Edinburgh.*—We learn from a correspondent in Edinburgh, that not fewer than *one hundred and sixty* gentlemen have just received the degree of M. D.

*St. Bartholomew's Hospital.*—Mr. EARLE has been elected one of the senior surgeons, on the resignation of Mr. ABERNETHY; and Mr. SKEY has been elected assistant surgeon.

*Middlesex Hospital.*—Mr. H. MAYO has been elected surgeon in the room of the late Mr. SHAW.

*St. George's Hospital.*—Our readers will learn with satisfaction that the building of the new hospital has at length commenced. It is so contrived as not to interfere with the present establishment; it will afford accommodation to a much larger number of patients; and, being a handsome elevation, will be a great improvement to that part of the town, the present building being, it must be acknowledged, any thing rather than ornamental.

### METEOROLOGICAL JOURNAL,

*From June 20th, to July 20th*, 1827.

By Messrs. HARRIS and Co. Mathematical Instrument Makers, 50, High Holborn.

| July | Rain gauge. | Moon. | Thermom. | | | Barometer. | | De Luc's Hygrom. | | Winds. | | Atmospheric Variations. | | |
|---|---|---|---|---|---|---|---|---|---|---|---|---|---|---|
| | | | 9 A.M. | MAX. | MIN. | 9 A.M. | 10 P.M. | 9 A.M. | 10 P.M. | 9 A.M. | 10 P.M. | 9 *a.m.* | 2 *p.m.* | 10 *p.m.* |
| 20 | | | 60 | 67 | 55 | 29.63 | 29.72 | 88 | 72 | W | W | Cloudy | Fair | Fine |
| 21 | | | 62 | 70 | 52 | 29.81 | 29.93 | 78 | 70 | WNW | WSW | — | — | — |
| 22 | | | 64 | 64 | 57 | 29.91 | 29.86 | 75 | 92 | SSW | SSW | — | Rain | Rain |
| 23 | .19 | | 66 | 74 | 64 | 29.88 | 29.96 | 93 | 88 | SSW | SSW | Rain | Cloudy | Fine |
| 24 | | | 66 | 73 | 62 | 29.96 | 29.91 | 92 | 88 | WSW | SW | Cloudy | — | — |
| 25 | | ● | 66 | 74 | 56 | 29.85 | 29.90 | 90 | 82 | WSW | SW | — | Rain | — |
| 26 | .28 | | 64 | 73 | 60 | 29.83 | 29.78 | 78 | 97 | SW | SW | — | — | Rain |
| 27 | | | 64 | 76 | 67 | 29.77 | 29.97 | 78 | 85 | NNW | SW | Fair | Fair | Fine |
| 28 | | | 70 | 78 | 64 | 30.08 | 30.09 | 88 | 77 | W | WNW | — | Fine | — |
| 29 | | | 76 | 84 | 67 | 29.99 | 29.87 | 82 | 85 | SE | E | — | — | — |
| 30 | | | 74 | 79 | 56 | 29.67 | 29.95 | 77 | 72 | W | WNW | — | — | — |
| 31 | | | 64 | 73 | 61 | 30.11 | 30.11 | 78 | 68 | W | NW | — | — | — |
| Aug. | | ☽ | | | | | | | | | | | | |
| 1 | | | 65 | 75 | 60 | 30.04 | 29.93 | 75 | 70 | SW | NW | — | — | — |
| 2 | | | 64 | 80 | 65 | 29.81 | 29.66 | 75 | 72 | SSE | SW | — | — | — |
| 3 | .26 | | 67 | 75 | 60 | 29.65 | 29.55 | 80 | 82 | SW | SW | — | Fair | Rain |
| 4 | .10 | | 64 | 73 | 61 | 29.54 | 29.76 | 84 | 76 | SW | W | Cloudy | Rain | Cloudy |
| 5 | | | 69 | 73 | 55 | 29.94 | 30.16 | 72 | 88 | WNW | NE | — | Fair | Rain |
| 6 | | | 57 | 68 | 53 | 30.16 | 30.20 | 90 | 86 | NE | E | Rain | — | Fine |
| 7 | | ○ | 60 | 69 | 52 | 30.18 | 30.10 | 77 | 72 | ESE | E | Fair | Fine | — |
| 8 | | | 57 | 72 | 55 | 30.03 | 29.95 | 77 | 78 | ENE | E | — | — | — |
| 9 | | | 62 | 70 | 50 | 29.90 | 29.80 | 82 | 76 | NE | SW | — | — | Fair |
| 10 | | | 53 | 73 | 56 | 29.56 | 29.54 | 86 | 71 | SW | W | Cloudy | — | — |
| 11 | .32 | | 60 | 69 | 55 | 29.48 | 29.50 | 74 | 80 | WSW | W | — | Rain | — |
| 12 | | | 61 | 68 | 52 | 29.57 | 29.77 | 82 | 67 | NW | NW | — | Fair | Fine |
| 13 | | | 59 | 67 | 60 | 29.79 | 29.69 | 75 | 88 | W | WSW | — | Cloudy | Rain |
| 14 | | ☾ | 65 | 70 | 63 | 29.56 | 29.35 | 93 | 88 | W | SW | — | Fair | Fair |
| 15 | | | 65 | 72 | 59 | 29.27 | 29.31 | 81 | 78 | W | SSW | — | — | Cloudy |
| 16 | .34 | | 62 | 69 | 56 | 29 31 | 29.45 | 88 | 93 | E | E | — | Rain | — |
| 17 | | | 62 | 69 | 57 | 29.63 | 29.81 | 88 | 92 | E | ENE | — | — | — |
| 18 | | | 61 | 69 | 55 | 29.86 | 29.91 | 92 | 85 | ENE | ENE | — | Fair | — |
| 19 | | | 62 | 68 | 55 | 29.87 | 29.92 | 74 | 82 | ENE | ENE | Fair | — | — |

The quantity of Rain fallen in the month of July, was 1 inch and 18.100ths.

